# Optimal treatment and prognostic factors for esthesioneuroblastoma: retrospective analysis of 187 Chinese patients

**DOI:** 10.1186/s12885-017-3247-z

**Published:** 2017-04-11

**Authors:** Le Xiong, Xiao-Li Zeng, Chang-Kuo Guo, An-Wen Liu, Long Huang

**Affiliations:** Department of Oncology, The Second Affiliated Hospital of Nanchang University, 1 Minde Road, Nanchang, Jiangxi Province 330006 China

**Keywords:** Esthesioneuroblastoma, Prognostic factors, Treatment

## Abstract

**Background:**

The standard treatment for esthesioneuroblastoma, a rare malignant nasal vault neoplasm, is not established.

**Methods:**

We retrospectively assessed the clinicopathological features, prognostic factors and treatment methods for 187 patients with esthesioneuroblastoma treated in China between 1981 and 2015. Overall survival (OS) and disease-free survival (DFS) were evaluated using the Kaplan-Meier method and log-rank tests.

**Results:**

Twenty-three (12.3%), 48 (25.7%) and 113 (60.4%) patients had Kadish stage A, B and C esthesioneuroblastoma; 3 (1.6%) had unknown stage. Overall, 117 (62.6%) patients received surgery and combined radiotherapy with or without chemotherapy; 35 (18.7%) received radiotherapy with or without chemotherapy; 32 (17.1%) received surgery alone; and 3 (1.6%) received palliative treatment. Three-year OS and DFS for the entire cohort were 66.7% and 57.5%, respectively. Three-year OS for stage A, B and C were 91.3%, 91.2% and 49.5% (*P <* 0.0001). Three-year OS was 16.7% and 66.7% for patients with and without distant metastasis (*P <* 0.0001). Surgery and combined radiotherapy with or without chemotherapy led to better OS and DFS than other treatment modes (both *P <* 0.0001). Univariate and multivariate analysis showed distant metastasis (hazard ratio [HR] = 2.162, 95% confidence interval [CI] = 1.145, 4.082, *P* = 0.017) and not receiving a combined modality treatment (HR = 2.391, 95% CI = 1.356, 4.218, *P* = 0.003) were independent prognostic factors for poor OS and DFS.

**Conclusions:**

This study indicates surgery and combined radiotherapy may currently be the optimal treatment for esthesioneuroblastoma.

**Electronic supplementary material:**

The online version of this article (doi:10.1186/s12885-017-3247-z) contains supplementary material, which is available to authorized users.

## Background

Esthesioneuroblastoma (ENB), also known as olfactory neuroblastoma, is a rare malignant neoplasm of the nasal vault that is believed to arise from neurosensory receptor cells in the olfactory epithelium [1, 2]. ENB accounts for 3% of all nasal tumors [3, 4]. The treatments for ENB include surgery, radiotherapy and/or chemotherapy [5–7], though it is difficult to achieve radical treatment using these strategies as most patients are diagnosed at a late stage. ENB is insidious and has a high propensity for invading adjacent organs and tissues. Distant metastasis mainly occurs via the lymph nodes and blood. The cervical lymph nodes [8], lungs, brain and bones are frequently reported sites of metastasis [9]. The limited number of patients and the long-time span have made it difficult to establish the features of this disease, such as its natural history, prognostic indicators, treatment techniques, and survival rates [10, 11]. For this study, we retrospectively assessed 187 patients with ENB treated in China. To the best of our knowledge, this is the first study in which treatment and prognostic factors have been assessed in a relatively large group of patients with ENB. The goal of this study was to help identify the clinical profile, treatment outcomes, and significant prognostic indicators in ENB.

## Methods

### Patient characteristics

A total of 187 ENB cases treated in China between 1981 and 2015 were retrospectively reviewed. Patients were eligible if they had a conclusive histopathologic diagnosis of ENB with complete clinical pathology, and no history of previous malignant disease or a second primary tumor. The median age was 37 years (range, 3–72 years). Median follow-up was 34 months (range, 1–204 months); 81 (43.3%) patients died or suffered recurrence during follow-up, and 4 patients died within 1 month of diagnosis. The study was carried out in accordance with relevant guidelines and regulations. All experimental protocols were approved by the medical ethics committee of the Second Affiliated Hospital of Nanchang University.

### Treatment

Primary treatment consisted primarily of surgery. Individualized postoperative treatment consisted of radiation therapy alone, chemotherapy alone, or concurrent chemoradiation therapy. Of the total of 187 patients, 117 (62.6%) received surgery and combined radiotherapy with or without chemotherapy; 35 (18.7%) received radiotherapy with or without chemotherapy; 32 (17.1%) received surgery alone; and 3 (1.6%) received palliative treatment only (Additional file 1: Table S1). The surgical approaches mainly include lateral rhinotomy, combined craniofacial resection, and endoscopic surgery. A total of 149 (79.7%) patients were managed with surgery: 94 (63.1%) by open surgery and 55 (36.9%) by endoscopic surgery. One hundred and six (71.1%) patients received gross-total resection, 31 (20.8%) received subtotal resection, and the surgical notes for 12 (8.1%) patients were unavailable. Radiation therapy was delivered to the tumor bed and local extension with nodal irradiation reserved for involved nodes. In most cases, radiation was combined with surgery, including pre-operative radiation therapy in 7 (4.6%) patients and post-operative radiation therapy in 110 (72.4%) patients, another 35 (23.0%) patients were treated with definitive radiation therapy. The radiation therapy doses varied from 60 to 70 Gy. Thirty-seven patients (19.8%) received chemotherapy, which consisted of etoposide and cisplatin, prednisone in the majority of patients; adriamycin, vincristine and cyclophosphamide were also used in some patients.

### Statistical analysis

Overall survival (OS) and disease-free survival (DFS) were evaluated using the Kaplan-Meier method and log-rank test. The Cox proportional hazards model was used to identify independent prognostic factors for OS and DFS. All analyses were carried out using SPSS software (version 17.0, SPSS Inc., Chicago, IL, USA). *P*-values <0.05 were considered to indicate statistical significance.

## Results

### Clinical features

A total of 187 patients were included in this study; 111 (59.4%) were male, 67 (35.8%) were female and data on sex was not available for 9 (4.8%) patients. According to the Kadish staging system, the distribution of patients with stage A, B and C esthesioneuroblastoma was 23 (12.3%), 48 (25.7%) and 113 (60.4%); data on stage was not available for 3 (1.6%) patients. All primary tumors were located in the nasal cavity with (*n* = 21) or without lymph node metastasis (*n* = 166), and with (*n* = 24) or without distant metastasis (*n* = 163). The sites of distant metastasis were the lungs, brain, bones and breast. The average time to recurrence was 15 months (range, 1–63 months). Sixty-two (33.2%) patients suffered recurrence, including 16 (25.8%) local recurrences, 9 (14.5%) lymph node recurrences, 22 (35.5%) distant recurrences, 9 (14.5%) cases of two types of recurrence, 1 (1.6%) case of all three types of recurrence and 5 (8.1%) recurrences for which complete data was not available.

### Survival outcomes

By last follow-up, sixty-two (33.2%) patients had suffered recurrence, including 16 (25.8%) local recurrences, nine (14.5%) lymph node recurrences, 22 (35.5%) distant recurrences, nine (14.5%) cases of two types of recurrence, 1 (1.6%) case of all three types of recurrence, and five (8.1%) recurrences for which complete data was not available. Sixty-two patients had died and 115 patients were still alive. Three-year overall survival (OS) and disease-free survival (DFS) were 66.7% and 57.5%, respectively.

### Prognostic factors

**Fig. 1:**
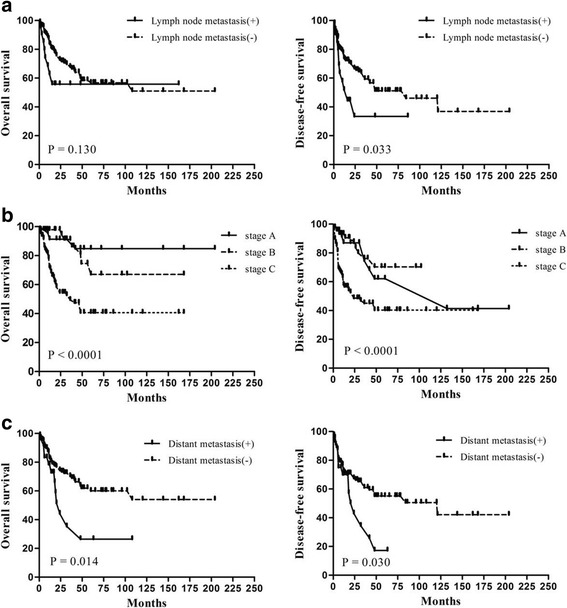
Kaplan-Meier OS (*left*) and DFS (*right*) curves for patients with ENB stratified by various clinicopathologic factors. **a** Survival curves for patients with and without lymph node metastasis; **b** for patients stratified by Kadish stage; and **c** for patients with and without distant metastasis

To identify potential prognostic factors associated with survival in ENB, various clinicopathologic variables were evaluated (Table 1). Univariate analysis identified stage, distant metastasis and treatment modalities were significantly associated with OS and DFS (*P* < 0.05), and lymph node metastasis was associated with DFS (*P* < 0.05) but not OS (*P* = 0.130; Fig. 1a). The 3-year OS rates for stage A, B and C ENB were 91.3%, 91.2% and 49.5%, respectively (*P <* 0.0001; Fig. 1b). The 3-year OS rates for patients with and without distant metastasis were 16.7% and 66.7%, respectively (*P <* 0.0001; Fig. 1c). Multivariate analysis showed distant metastasis (hazard ratio [HR] = 2.162, 95% confidence interval [CI] = 1.145, 4.082, *P* = 0.017) and not receiving a combined modality treatment (HR = 2.391, 95% CI = 1.356, 4.218, *P* = 0.003) were independent prognostic factors for poor OS and DFS.

**Table 1:** Three-year OS and DFS rates for ENB

Characteristic	Total *n* (%)	3-year		3-year	
		DFS	*P*	OS	*P*
Gender					
Male	111 (64.2)	58.4%		66.1%	
Female	67 (37.6)	57.0%	0.640	64.7%	0.366
Lymph node metastasis					
(+)	21 (11.2)	33.4%		55.8%	
(−)	166 (88.8)	59.4%	**0.033**	67.8%	0.130
Distant metastasis					
(+)	24 (12.8)	34.4%		35.3%	
(−)	163 (87.2)	60.9%	**0.030**	70.3%	**0.014**
Stage					
A	23 (12.5)	74.5%		91.3%	
B	48 (26.1)	76.1%		91.2%	
C	113 (61.4)	45.0%	**0.000**	49.5%	**0.000**
Treatment					
Surgery					
Yes	148 (79.1)	58.9%		72.0%	
No	39 (20.9)	47.4%	**0.038**	45.7%	**0.000**
Extent of resection					
Gross total	106 (77.4)	72.5%		90.1%	
Subtotal	31 (22.6)	31.4%	**0.000**	46.8%	**0.000**
RT					
Yes	153 (81.8)	65.4%		70.8%	
No	34 (18.2)	22.7%	**0.000**	49.1%	**0.021**
CT					
Yes	37 (19.8)	67.4%		69.1%	
No	150 (80.2)	54.3%	0.162	66.0%	0.472
S + RT ± CT					
Yes	117 (62.6)	69.0%		77.1%	
No	70 (37.4)	37.1%	**0.000**	49.8%	**0.000**
S + RT					
Yes	88 (47.1)	66.5%		76.0%	
No	99 (52.9)	47.8%	**0.019**	57.9%	**0.011**
S + RT ± CT					
S + RT	88 (75.2)	66.5%		76.0%	
S + RT + CT	29 (24.8)	75.3%	0.283	80.6%	0.589
Recurrence					
Yes	62 (33.2)	14.1%		41.0%	
No	125 (66.8)	81.6%	**0.000**	82.8%	**0.000**

*S* surgery, *CT* chemotherapy, *RT* radiotherapy, Bold indicates significant values

**Fig. 2:**
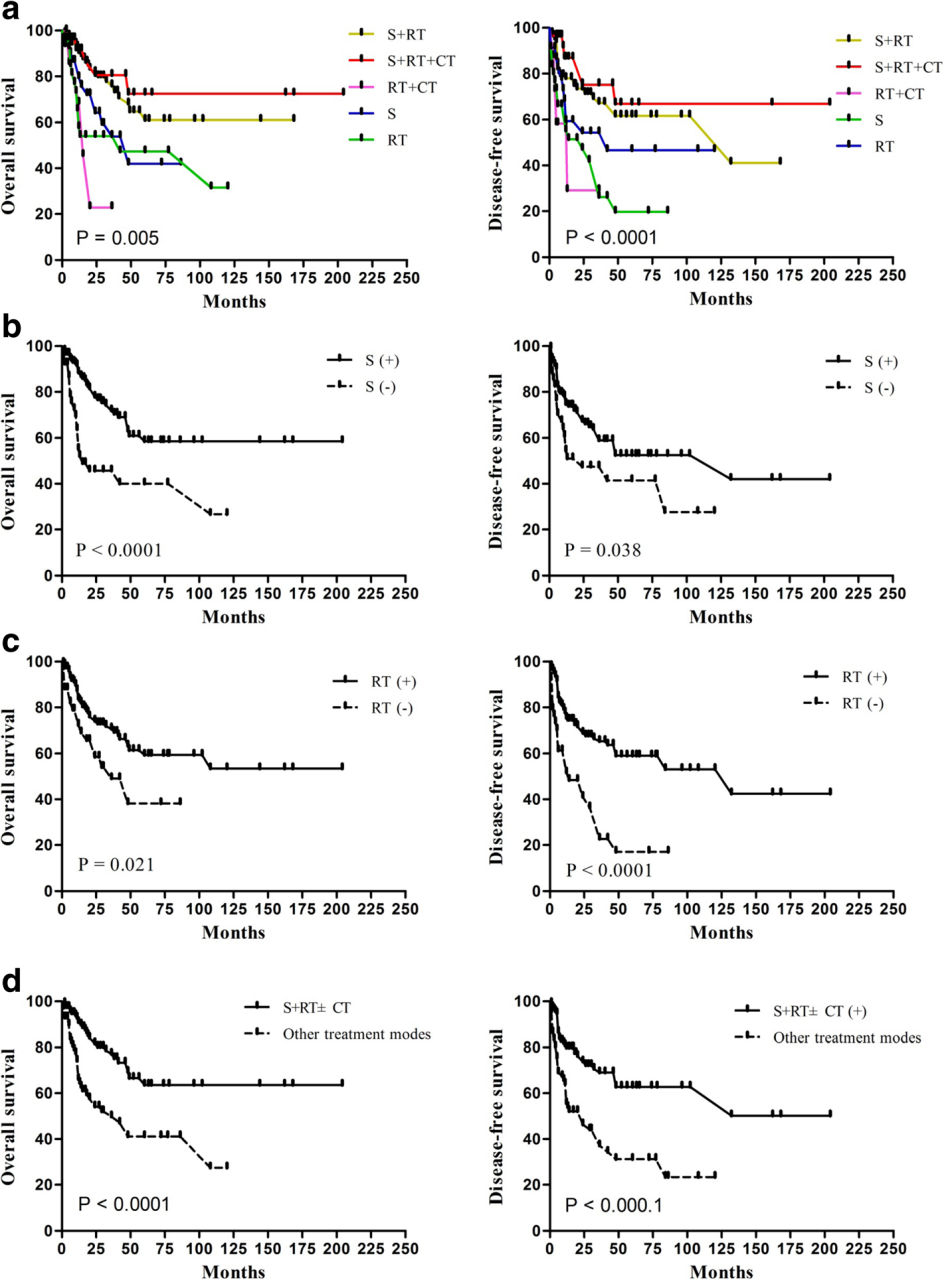
Kaplan-Meier OS (*left*) and DFS (*right*) curves for patients with ENB stratified by treatment. **a** Survival curves for each treatment; **b** for treatments including surgery compared with other treatments; and **c** for treatments including radiotherapy compared with other treatments; and **d** Survival curves for surgery and combined radiotherapy with or without chemotherapy compared to other treatment modes

The 3-year OS rates for patients who received surgery alone, radiotherapy alone, surgery combined with radiotherapy, surgery combined with radiotherapy and chemotherapy, and surgery combined with chemotherapy were 56.3%, 52.2%, 75.6%, 80.6%, and 22.9%, respectively (Fig. 2a). Overall, patients who received surgery (including surgery alone, surgery combined with radiotherapy, surgery combined with radiotherapy and chemotherapy) achieved significantly better OS (*P* = 0.0002) and DFS (*P* = 0.0376) than patients receiving other treatment modes (Fig. 2b). Radiotherapy (including radiotherapy alone, surgery combined with radiotherapy, surgery combined with radiotherapy and chemotherapy, radiotherapy and chemotherapy) resulted in significantly better OS (*P* = 0.0211) and DFS (*P* = 0.0000) compared to surgery alone and other treatment modes (Fig. 2c). Chemotherapy (including surgery combined with radiotherapy and chemotherapy, radiotherapy and chemotherapy) did not result in significantly better OS (*P* = 0.4723) or DFS (*P* = 0.1624) compared to surgery alone, radiotherapy alone or surgery combined with radiotherapy.

Furthermore, surgery and combined radiotherapy with or without chemotherapy resulted in significantly better OS and DFS (both *P* = 0.000) compared with other treatment modes (Fig. 2d). Surgery and combined radiotherapy with chemotherapy did not result in significantly better OS (*P* = 0.589) or DFS (*P* = 0.283) compared to surgery and combined radiotherapy. These results indicate that surgery and combined radiotherapy may represent the standard treatment for ENB.

## Discussion

Due to its rarity, there is limited data on ENB in the literature. Before diagnosis, the most common clinical symptoms include nasal obstruction, epistaxis and hyposmia. Though the patterns of spread are relatively well-characterized: tumor metastasis occurs in mid- and late stage disease via the lymph nodes and blood, much remains to be learned about ENB [12]. Few studies have systematically evaluated the treatment methods for this tumor type, thus there is no general consensus on the optimal therapeutic approach [13]. Therefore, we retrospectively assessed the clinicopathological features, prognostic factors and treatment methods for a series of 187 Chinese patients with ENB. As the patients were treated over a long time-span, we staged the patients using the original Kadish staging system.

Some of the basic characteristics of this study population, such as the median age of 37 years and large age range, are in accordance with previously published studies (refs), although one study reported a median age of 27 years for 21 patients [14]. Some of inadequacies of the Kadish system include inability to effectively stratify patients, with few patients falling into group A and several different types of spread being consolidated in group C [15, 16]. However, we found this staging system had acceptable prognostic utility. The distribution of patients with stage A, B and C esthesioneuroblastoma according to the Kadish staging system was 23 (12.3%), 48 (25.7%) and 113 (60.4%) in this cohort.

This study indicates that distant metastasis, the treatment modality and stage were significantly associated with OS and DFS in ENB, while lymph node metastasis was associated with DFS, but not OS. These findings contradict previous data on the relationship between lymph node metastasis in ENB and OS [1]. The literature reports a 10% to 33% incidence of lymph node metastasis, which is similar to the rate of 11.2% in this cohort. Lymph node metastasis had a clear impact on disease-specific survival in the present study, consistent with other reports [17, 18]. Elkon et al. [19] found a favorable 3-year overall survival rate in patients with stage A or B disease (88.9% and 83.3%, respectively), while patients with stage C disease had 3-year survival of only 52.9%. Similarly, 3-year OS for stage A, B and C were 91.3%, 91.2% and 49.5%, respectively, in this study. Unfortunately, the short median follow-up of 34 months is a limitation; only 15.5% of patients had more than 5 years of follow-up. We chose to examine 3-year survival rather than 5-year survival, which is more commonly reported in the literature; this makes comparison of our results with other studies more difficult.

Over the last several decades, it has become clear that ENB must be treated aggressively and systemically, although reports exist of patients being cured with local radiotherapy alone. Our data revealed improved survival (3-year OS: 81.5% vs. 56.3%) with multimodality treatment (surgery and radiotherapy) compared to radiotherapy alone, consistent with previous studies (refs). Our analysis indicates surgery is beneficial, but selection bias can exist in any retrospective review. For example, patients with wider infringement of lesions may be given the choice of radiotherapy or chemotherapy.

The impact of chemotherapy on this disease cannot be determined in this analysis, as the cohort were treated over a long period of time, during which treatment options and diagnostic techniques evolved. Further studies are required to determine whether chemotherapy is necessary, consistent with previous studies [19–22]. Moreover, the value of neoadjuvant chemoradiotherapy was not assessed in this study due to the small number of patients (only seven) receiving this treatment modality, limiting our ability to make meaningful comparisons with this subgroup, though previous studies have shown neoadjuvant chemoradiotherapy provides a survival benefit [23–25]. After reviewing our data and the literature, we suggest that combined modality treatment (surgery and radiotherapy) may significantly decrease recurrence and improve OS and DFS in ENB.

## Conclusions

We acknowledge that this study has the limitations of retrospective data collection and analysis of multi-center experience over a long period of 34 years. However, given the low incidence of ENB, this study had a large sample size. Notwithstanding its limitations, we believe the current analysis provides evidence to recommend surgery and combined radiotherapy as the current optimal treatments for ENB and may assist validation studies of larger and prospective data sets.

## Additional files

Additional file 1: Table S1. Clinical and pathologic features of patients with ENB treated in China. (DOCX 50 kb)

## Abbreviations

CI: Confidence interval
CT: Chemotherapy
DFS: Disease-free survival
ENB: Esthesioneuroblastoma
HR: Hazard ratio
OS: Overall survival
RT: Radiotherapy
S: Surgery
