# Supplementary material for: Optimal treatment and prognostic factors for esthesioneuroblastoma: retrospective analysis of 187 Chinese patients
Source: BMC Cancer. 2017 Apr 11;17:254. doi: 10.1186/s12885-017-3247-z (PMC5387340; doi:10.1186/s12885-017-3247-z)
Supplement: Supplementary file 1 — Clinical and pathologic features of patients with ENB treated in China. (DOCX 50 kb) [file 12885_2017_3247_MOESM1_ESM.docx]

| Table S1. The clinical and pathologic features of patients with ENB examined in china. | | | | | | | | | |
| --- | --- | --- | --- | --- | --- | --- | --- | --- | --- |
| Case | Age range(year) | Interval | Lymph node metastasis | Distant metastasis | Stage | **Therapy** | **Recurrence** | | **Follow-up** |
| number |  | (Onset-diagnosis) |  |  |  |  | **Interval** | **Site** | **(month)** |
| 1 | 20-40 | 1 | (+) | (-) | C | S+RT | (-) | (-) | 7 |
| 2 | 40-60 | ___ | (-) | (-) | B | S+RT | (-) | (-) | 56 |
| 3 | 20-40 | ___ | (-) | (-) | B | S+RT | (-) | (-) | 40 |
| 4 | 60-80 | 9 | (-) | (-) | C | S | 6mo | LR | 48 |
| 5 | 0-20 | 8 | (-) | (-) | C | S+RT+CT | (-) | (-) | 48 |
| 6 | 0-20 | 5 | (-) | (-) | C | S | 3mo | LR | 6 |
| 7 | 0-20 | 10 | (-) | (-) | B | S | 4mo | LR | 48 |
| 8 | 0-20 | 8 | (-) | (-) | C | S+RT | ND | LNR | 12 |
| 9 | 0-20 | 6 | (-) | (-) | C | S | (-) | (-) | 12 |
| 10 | 60-80 | 2 | (-) | (-) | B | S | (-) | (-) | 72 |
| 11 | 20-40 | 6 | (-) | (-) | B | S+RT+CT | (-) | (-) | 60 |
| 12 | 40-60 | 8 | (-) | (-) | B | S+RT | 36mo | LR | 60 |
| 13 | 0-20 | 4 | (-) | (-) | A | S+RT | 48mo | LNR | 96 |
| 14 | 20-40 | 5 | (-) | (-) | B | S+RT | (-) | (-) | 36 |
| 15 | 40-60 | 3 | (-) | (-) | B | S+RT | (-) | (-) | 60 |
| 16 | 20-40 | 2 | (-) | (-) | B | S+RT | (-) | (-) | 36 |
| 17 | 40-60 | 10 | (-) | (-) | C | S+RT | (-) | (-) | 48 |
| 18 | 40-60 | 1 | (-) | (-) | C | S | 2mo | LR | 48 |
| 19 | 60-80 | 3 | (-) | (-) | B | S+RT | (-) | (-) | 60 |
| 20 | 40-60 | 2 | (-) | (-) | C | S+RT+CT | (-) | (-) | 36 |
| 21 | 20-40 | 9 | (-) | (-) | B | S+RT | 7mo | LNR | 36 |
| 22 | 40-60 | 70 | (-) | (-) | B | S+RT | (-) | (-) | 36 |
| 23 | 40-60 | 15 | (-) | (-) | C | S+RT | (-) | (-) | 24 |
| 24 | 20-40 | 3 | (-) | (-) | C | S+RT+CT | (-) | (-) | 6 |
| 25 | 40-60 | 6 | (-) | (-) | B | S+RT+CT | (-) | (-) | 48 |
| 26 | 40-60 | 12 | (-) | (-) | B | S | (-) | (-) | 24 |
| 27 | 40-60 | 36 | (-) | (-) | C | RT | (-) | (-) | 17 |
| 28 | 20-40 | 1 | (-) | (-) | C | RT+CT | (-) | (-) | 12 |
| 29 | 20-40 | 12 | (-) | (-) | B | S+RT+CT | (-) | (-) | 9 |
| 30 | 40-60 | 10 | (-) | (-) | B | S+RT+CT | (-) | (-) | 8 |
| 31 | 60-80 | 18 | (-) | (-) | B | S+RT+CT | (-) | (-) | 5 |
| 32 | 0-20 | ___ | (-) | (-) | C | S | (-) | (-) | 20 |
| 33 | 20-40 | 6 | (-) | (-) | A | S+RT+CT | (-) | (-) | 204 |
| 34 | 40-60 | 6 | (-) | (-) | C | S+RT | (-) | (-) | 24 |
| 35 | 40-60 | 12 | (+) | (-) | B | S+RT | (-) | (-) | 19 |
| 36 | 60-80 | 1 | (-) | (-) | C | S | (-) | (-) | 24 |
| 37 | 40-60 | 36 | (-) | (-) | A | S | (-) | (-) | 20 |
| 38 | 20-40 | 12 | (-) | (-) | C | S | 6mo | LR | 14 |
| 39 | 60-80 | 3 | (-) | (-) | B | S+RT | (-) | (-) | 18 |
| 40 | 0-20 | 1 | (+) | (-) | C | S+RT | (-) | (-) | 12 |
| 41 | 40-60 | ___ | (-) | (-) | C | S+RT+CT | (-) | (-) | 48 |
| 42 | 60-80 | ___ | (-) | (-) | C | S+RT+CT | (-) | (-) | 12 |
| 43 | 20-40 | 24 | (-) | (-) | ___ | S | (-) | (-) | 144 |
| 44 | 20-40 | 24 | (-) | (-) | ___ | S+RT | NM | NM | 107 |
| 45 | 40-60 | ___ | (-) | (+) | C | S+RT | (-) | (-) | 24 |
| 46 | 20-40 | ___ | (-) | (-) | A | S+RT | (-) | (-) | 24 |
| 47 | 40-60 | ___ | (-) | (+) | C | RT | (-) | (-) | 9 |
| 48 | 20-40 | ___ | (-) | (+) | C | S+RT | (-) | (-) | 18 |
| 49 | 0-20 | ___ | (-) | (+) | C | ST | (-) | (-) | 6 |
| 50 | 40-60 | ___ | (-) | (+) | C | RT | (-) | (-) | 11 |
| 51 | 20-40 | ___ | (-) | (-) | B | S+RT | (-) | (-) | 24 |
| 52 | ___ | ___ | (-) | (-) | B | S+RT | NM | LR+LNR | 49 |
| 53 | ___ | ___ | (-) | (-) | B | S+RT | NM | LR+Bone | 38 |
| 54 | ___ | ___ | (-) | (-) | B | S+RT | (-) | (-) | 102 |
| 55 | ___ | ___ | (-) | (-) | C | S+RT | (-) | (-) | 74 |
| 56 | ___ | ___ | (-) | (-) | C | S+RT | NM | LR+LNR+Brain | 41 |
| 57 | ___ | ___ | (-) | (-) | C | RT | NM | Lungs | 11 |
| 58 | ___ | ___ | (-) | (-) | C | RT | NM | Lungs | 13 |
| 59 | ___ | ___ | (-) | (-) | C | RT | (-) | (-) | 77 |
| 60 | ___ | ___ | (-) | (-) | C | RT | NM | Lungs+Liver | 6 |
| 61 | 40-60 | ___ | (-) | (-) | C | RT | (-) | (-) | 108 |
| 62 | 40-60 | ___ | (-) | (-) | C | RT+CT | 36mo | LNR | 36 |
| 63 | 40-60 | 12 | (+) | (+) | C | S+RT | (-) | (-) | 18 |
| 64 | 20-40 | ___ | (-) | (-) | C | S+RT+CT | 11mo | Breast | 17 |
| 65 | 0-20 | 10 | (-) | (-) | B | S | 14mo | LR | 30 |
| 66 | 0-20 | 3 | (-) | (-) | B | S | (-) | (-) | 29 |
| 67 | 40-60 | 3 | (-) | (-) | B | S+RT | (-) | (-) | 36 |
| 68 | 60-80 | ___ | (-) | (-) | B | S+RT | (-) | (-) | 96 |
| 69 | 20-40 | 12 | (+) | (-) | C | ST | (-) | (-) | 2 |
| 70 | 20-40 | 12 | (-) | (-) | B | S+RT | (-) | (-) | 26 |
| 71 | 20-40 | 60 | (-) | (-) | A | S+RT | (-) | (-) | 48 |
| 72 | 40-60 | 1 | (+) | (-) | C | RT+CT | (-) | (-) | 3 |
| 73 | 40-60 | 5 | (-) | (-) | C | S+RT+CT | (-) | (-) | 9 |
| 74 | 20-40 | 84 | (-) | (-) | C | S | (-) | (-) | 4 |
| 75 | 60-80 | 12 | (-) | (-) | C | RT | (-) | (-) | 120 |
| 76 | 40-60 | 3 | (-) | (-) | B | RT | (-) | (-) | 36 |
| 77 | 40-60 | 5 | (-) | (+) | C | S+RT | (-) | (-) | 12 |
| 78 | 40-60 | 2 | (+) | (-) | C | RT | (-) | (-) | 3 |
| 79 | 20-40 | 34 | (-) | (-) | B | RT | (-) | (-) | 60 |
| 80 | 20-40 | ___ | (-) | (-) | A | S+RT | (-) | (-) | 48 |
| 81 | 60-80 | ___ | (-) | (-) | A | S+RT | (-) | (-) | 24 |
| 82 | 20-40 | ___ | (-) | (-) | C | S+RT | 6mo | LR | 7 |
| 83 | 0-20 | 1.5 | (-) | (+) | C | S | (-) | (-) | 9 |
| 84 | 20-40 | 0.7 | (+) | (+) | C | S+RT+CT | (-) | (-) | 48 |
| 85 | 20-40 | 6 | (-) | (+) | C | S+RT | 18mo | LR+Brain | 19 |
| 86 | 20-40 | 0.3 | (-) | (+) | C | S+RT | (-) | (-) | 16 |
| 87 | 20-40 | 6 | (-) | (+) | C | S+RT | 20mo | Brain | 20 |
| 88 | 60-80 | 2 | (-) | (+) | C | S | NM | NM | 2 |
| 89 | 20-40 | 1 | (-) | (+) | C | S+RT | 32mo | Bone | 32 |
| 90 | 40-60 | 36 | (-) | (+) | C | S+RT | 48mo | NM | 48 |
| 91 | 40-60 | 1 | (-) | (-) | C | S+RT+CT | (-) | (-) | 52 |
| 92 | 40-60 | 0.3 | (-) | (-) | C | S+RT+CT | (-) | (-) | 46 |
| 93 | 20-40 | 6 | (-) | (+) | C | S+RT+CT | 24mo | NM | 24 |
| 94 | 0-20 | 12 | (-) | (-) | C | S+RT+CT | (-) | (-) | 14 |
| 95 | 20-40 | 12 | (-) | (+) | C | S+RT | 63mo | LNR | 63 |
| 96 | 60-80 | ___ | (-) | (-) | B | S+RT | (-) | (-) | 2 |
| 97 | 20-40 | 7 | (+) | (-) | C | S+RT | 6mo | Breast | 12 |
| 98 | 20-40 | 24 | (-) | (-) | B | S+RT+CT | (-) | (-) | 6 |
| 99 | 60-80 | ___ | (-) | (-) | B | S+RT | (-) | (-) | 24 |
| 100 | 40-60 | ___ | (-) | (-) | B | RT | (-) | (-) | 24 |
| 101 | 40-60 | ___ | (-) | (-) | B | S+RT | 10mo | Bone | 10 |
| 102 | 40-60 | ___ | (-) | (-) | B | S+RT+CT | (-) | (-) | 7 |
| 103 | 20-40 | ___ | (-) | (-) | C | RT | (-) | (-) | 4 |
| 104 | 40-60 | 8 | (-) | (-) | B | S+RT | (-) | (-) | 60 |
| 105 | 0-20 | 2 | (-) | (-) | C | S | (-) | (-) | 2 |
| 106 | 40-60 | 24 | (-) | (-) | C | S+RT+CT | (-) | (-) | 65 |
| 107 | 20-40 | 3 | (-) | (-) | C | S+RT | 6mo | Brain | 36 |
| 108 | 20-40 | 3 | (-) | (-) | C | S+RT | 6mo | Brain | 14 |
| 109 | 20-40 | 24 | (-) | (-) | B | S+RT+CT | 24mo | LR | 168 |
| 110 | 60-80 | 6 | (-) | (-) | C | RT | 12mo | LR | 12 |
| 111 | 20-40 | 1.5 | (-) | (-) | C | RT | (-) | (-) | 36 |
| 112 | 20-40 | 6 | (-) | (-) | B | S+RT+CT | (-) | (-) | 24 |
| 113 | 40-60 | 2 | (-) | (-) | C | S+RT | (-) | (-) | 78 |
| 114 | 40-60 | 2 | (-) | (-) | C | S+RT | (-) | (-) | 60 |
| 115 | 0-20 | 2 | (-) | (-) | C | S | (-) | (-) | 12 |
| 116 | 40-60 | 3 | (-) | (-) | C | S | (-) | (-) | 6 |
| 117 | 40-60 | 2 | (-) | (-) | B | S+RT | (-) | (-) | 12 |
| 118 | 20-40 | 3 | (-) | (-) | B | S+RT | (-) | (-) | 48 |
| 119 | 40-60 | ___ | (-) | (+) | C | S+RT | 6mo | LR+LNR | 12 |
| 120 | 20-40 | 5 | (-) | (-) | C | RT | 5mo | Bone | 5 |
| 121 | 0-20 | 1 | (+) | (-) | C | RT | 10mo | Brain | 10 |
| 122 | 20-40 | 6 | (-) | (-) | A | S | 36mo | LR | 72 |
| 123 | 20-40 | 60 | (-) | (-) | A | S | (-) | (-) | 24 |
| 124 | 20-40 | 4 | (-) | (-) | A | S+RT | (-) | (-) | 12 |
| 125 | 0-20 | 7 | (-) | (-) | C | S+RT | 8mo | LR | 24 |
| 126 | 20-40 | 36 | (-) | (-) | C | S+RT | (-) | (-) | 36 |
| 127 | 20-40 | 0.7 | (-) | (-) | C | RT+CT | 13mo | Brain | 20 |
| 128 | 40-60 | 6 | (-) | (-) | A | S+RT | (-) | (-) | 24 |
| 129 | 20-40 | 24 | (-) | (-) | C | S | 36mo | LR+Brain | 36 |
| 130 | 40-60 | 1 | (-) | (-) | C | S | 2mo | LR+Brain | 8 |
| 131 | 0-20 | 1.7 | (+) | (-) | C | S+RT+CT | (-) | (-) | 24 |
| 132 | 40-60 | 1 | (-) | (+) | C | S+RT+CT | (-) | (-) | 6 |
| 133 | 40-60 | 2 | (-) | (-) | C | S+RT | (-) | (-) | 5 |
| 134 | 20-40 | 0.5 | (-) | (-) | C | S+RT | (-) | (-) | 36 |
| 135 | 20-40 | 6 | (-) | (-) | C | S+RT | (-) | (-) | 12 |
| 136 | 20-40 | 2 | (-) | (-) | B | S+RT | (-) | (-) | 18 |
| 137 | 60-80 | 6 | (-) | (-) | C | S+RT | (-) | (-) | 12 |
| 138 | 40-60 | 7 | (-) | (-) | A | S+RT | (-) | (-) | 24 |
| 139 | 20-40 | 6 | (-) | (-) | B | S+RT | (-) | (-) | 48 |
| 140 | 0-20 | 3 | (-) | (-) | ___ | S | 48mo | Brain | 24 |
| 141 | 40-60 | 1 | (-) | (-) | B | S | (-) | (-) | 48 |
| 142 | 20-40 | 8 | (-) | (-) | C | S+RT | 6mo | NM | 15 |
| 143 | 20-40 | 7 | (+) | (-) | C | S | (-) | (-) | 86 |
| 144 | 20-40 | 3 | (-) | (-) | A | S+RT | (-) | (-) | 52 |
| 145 | 40-60 | 2 | (-) | (+) | C | S+RT+CT | (-) | (-) | 17 |
| 146 | 40-60 | 1 | (+) | (-) | C | RT+CT | 4mo | LNR | 15 |
| 147 | 0-20 | 6 | (+) | (-) | C | RT+CT | (-) | (-) | 6 |
| 148 | 20-40 | 6 | (+) | (-) | C | RT | 12mo | LNR+Lungs | 12 |
| 149 | 20-40 | 36 | (-) | (-) | A | S | 36mo | EM | 48 |
| 150 | 0-20 | 7 | (-) | (-) | C | RT+CT | 3mo | EM | 12 |
| 151 | 20-40 | 6 | (+) | (-) | C | RT | 6mo | Bone | 6 |
| 152 | 20-40 | 2 | (+) | (+) | C | RT+CT | (-) | (-) | 5 |
| 153 | 60-80 | 5 | (-) | (-) | A | RT | (-) | (-) | 30 |
| 154 | 20-40 | 6 | (-) | (-) | B | S+RT | (-) | (-) | 36 |
| 155 | 0-20 | 24 | (-) | (-) | C | S+RT | (-) | (-) | 24 |
| 156 | 40-60 | 18 | (-) | (-) | A | RT | (-) | (-) | 60 |
| 157 | 20-40 | 60 | (-) | (-) | A | S+RT | (-) | (-) | 60 |
| 158 | 60-80 | 2 | (-) | (-) | A | RT | (-) | (-) | 42 |
| 159 | 20-40 | 120 | (+) | (-) | C | RT | (-) | (-) | 36 |
| 160 | 0-20 | 3 | (-) | (-) | C | RT | 1mo | EM | 7 |
| 161 | 40-60 | 48 | (-) | (-) | A | S+RT | (-) | (-) | 60 |
| 162 | 0-20 | 12 | (-) | (-) | A | S+RT | (-) | (-) | 12 |
| 163 | 0-20 | 1.5 | (-) | (-) | C | ST | (-) | (-) | 1 |
| 164 | 0-20 | 8 | (-) | (-) | A | S+RT+CT | 10mo | EM | 10 |
| 165 | 20-40 | 8 | (-) | (-) | C | S | 2mo | LNR+Breast | 27 |
| 166 | 20-40 | 8 | (-) | (-) | C | S+RT | (-) | (-) | 30 |
| 167 | 0-20 | 24 | (-) | (-) | C | S+RT | (-) | (-) | 28 |
| 168 | 20-40 | 0.5 | (-) | (-) | C | S+RT | 3mo | LR | 11 |
| 169 | 40-60 | ___ | (-) | (-) | C | S+RT | (-) | (-) | 168 |
| 170 | 20-40 | ___ | (-) | (+) | C | S | 42mo | LR | 9 |
| 171 | 0-20 | 1 | (+) | (-) | C | S+RT+CT | 16mo | Abdomina | 162 |
| 172 | 0-20 | 2 | (-) | (-) | C | S+RT | 6mo | Bone | 6 |
| 173 | 0-20 | 12 | (-) | (-) | C | RT | (-) | (-) | 60 |
| 174 | 60-80 | 6 | (-) | (-) | A | S+RT | (-) | (-) | 48 |
| 175 | 20-40 | 2 | (-) | (+) | C | S+RT | 6mo | Brain | 6 |
| 176 | 40-60 | 2 | (-) | (-) | B | S+RT | (-) | (-) | 6 |
| 177 | 60-80 | 36 | (-) | (-) | B | S+RT | (-) | (-) | 24 |
| 178 | 20-40 | 24 | (-) | (-) | B | S+RT | (-) | (-) | 12 |
| 179 | 20-40 | 3 | (-) | (-) | C | S+RT | 4mo | LNR | 4 |
| 180 | 40-60 | 2 | (-) | (-) | C | S | 12mo | LR+Brain | 18 |
| 181 | 20-40 | 1 | (-) | (-) | C | S+RT | 2mo | LR | 3 |
| 182 | 20-40 | 2 | (+) | (-) | C | S+RT+CT | 1mo | Brain | 3 |
| 183 | 20-40 | 7 | (-) | (-) | C | S+RT | (-) | (-) | 6 |
| 184 | 60-80 | 3 | (-) | (-) | C | RT | (-) | (-) | 1 |
| 185 | 20-40 | 5 | (-) | (-) | A | S+RT | (-) | (-) | 168 |
| 186 | 40-60 | 6 | (-) | (+) | C | S+RT | 5mo | LNR | 12 |
| 187 | 0-20 | 1 | (-) | (-) | C | S | 5mo | LR | 6 |
| S:Surgery;RT:Radiotherapy;CT:Chemotherapy;LR:Local Recurrence;LNR:Lymph Node Recurrence;EM:Extensive Metastasis;NM:Not Mentioned. | | | | | | | | | |
